# A simple cell proliferation assay and the inflammatory protein content show significant differences in human plasmas from young and old subjects

**DOI:** 10.3389/fbioe.2024.1408499

**Published:** 2024-09-09

**Authors:** A. Muraglia, O. Utyro, M. Nardini, M. Santolini, D. Ceresa, V. Agostini, A. Nencioni, G. Filaci, R. Cancedda, M. Mastrogiacomo

**Affiliations:** ^1^ Dipartimento di Medicina Interna e Specialità Mediche (DIMI), Università Degli Studi di Genova, Genova, Italy; ^2^ IRCCS Ospedale Policlinico San Martino, Genova, Italy; ^3^ Dipartimento di Medicina Sperimentale (DIMES), Università Degli Studi di Genova, Genova, Italy

**Keywords:** rejuvenation, young plasma injection, fetal foreskin fibroblast cell line HFFF2, breast cancer cell line MDA, cell proliferation support, senescence-associated secretory phenotype

## Abstract

Some studies showed a “rejuvenating” effect of exposing aging tissues to a young environment. In mouse heterochronic parabiosis experiments, in response to young organisms, old animals lived longer than isochrony old age-matched conjoint animals. Comparable “rejuvenating” effects were obtained by injecting young plasma in old mice. This raised great hopes of slowing down the senescence process in humans by the injection of young plasma, as well as to prevent or cure age-related diseases. Some clinical trials are currently being performed or were recently completed. However, these studies are small and of limited duration, and we still lack convincing evidence to support the effectiveness of young plasma injection. It is urgent to perform additional investigations, including the development of an assay to measure the cell proliferation induction capability of different human plasmas, before one can seriously think of a large-scale treatment of humans. We adopted a simple method to measure the potential of different plasmas in supporting cell line proliferation, regardless of the co-presence of a platelet lysate. By comparing plasmas from young and old subjects, we observed a decreased activity in plasmas from old individuals. The young plasma effect may be attributed to specific proteins and growth factors more abundant in younger individuals that could decrease with age. Alternatively, or at the same time, the reduced cell proliferation support could be due to inhibitors present in the old plasma. Studying the different protein content of young and old plasmas was out of the scope of this article. Such differences should be adequately investigated by proteomics using many samples. However, a preliminary study of the different protein content of young and old plasmas was part of the assay validation using a commercially available cytokine array for parallel determination of the relative levels of 105 selected human proteins. We could show the existence of specific differences between young and old plasmas and that plasmas from old individuals presented a higher concentration of “inflammatory” proteins.

## Introduction: the “rejuvenating” effect of young plasma

### Young blood plasma was shown to reduce the impact of aging in mice

The body tissue aging is linked to a progressive loss of homeostasis and regenerative potential of the organism. Some studies showed a “rejuvenating” effect of exposing aging tissues to a young environment.

In the early 1970s, scientists began to join rodents of different ages (heterochronic parabiosis) and discovered that, in response to the young organisms, the old animals lived longer compared to isochrony old age-matched conjoint animals. It was hypothesized that systemic alterations in the global regulators of tissue physiology present in the blood may coordinate this process. Conboy et al. showed that the rejuvenating effect of heterochronic parabiosis on skeletal muscle could be recapitulated *in vitro* by exposing aging satellite cells of mice to young serum (Conboy et al., 2005). On the contrary, the deleterious effect of an aged environment on stem cell functionality was observed in parabiotic pairings (Villeda et al., 2011). The same Stanford University group, by the injection of young plasma in mice, obtained an effect comparable to heterochronic parabiosis in reversing age-related weakening of cognitive function in spatial–memory tests (Villeda et al., 2014). The blood of young mice appeared to rejuvenate all organs of the old mice, making the animals healthier and cognitively fit. A more recent report showed a beneficial effect of young animal plasmas *in vivo* in a mouse model of Alzheimer's disease (Zhao et al., 2020). Finally, human umbilical cord blood plasma was shown to revitalize hippocampal function in aged mice (Castellano et al., 2017) and to promote neurogenesis while reducing inflammation in a rat model of ischemic stroke (Yoo et al., 2016).

### Treatment of humans with young plasma. Controversial evaluation of existing data

The discovery of tissue rejuvenation as a consequence of the transfusion of blood from young to aged animals raised incredible hopes of slowing down the senescence process in humans, as well as to prevent or cure age-related diseases such as cancer, brittle bones, and Alzheimer’s. However, obtaining the same results in humans is far from straightforward, and uncertainties still exist in a major part of the scientific and medical community. A recent publication reported the results of a randomized clinical trial performed to assess the safety, tolerability, and feasibility of four weekly infusions of young, fresh, frozen plasma (donors aged 18–30 years) to treat Alzheimer’s disease (AD) symptoms in 18 individuals aged 50–90 years (mean age 74 years) (Sha et al., 2019). Although no amelioration of neurological symptoms could be determined, the study showed no adverse events, indicating that plasma therapy was safe and well tolerated at least under a short-term protocol. Although most results were not significant, two metrics–the Functional Activities Questionnaire and the Alzheimer’s Disease Cooperative Study–Activities of Daily Living Inventory–showed a significant improvement, following plasma treatment. However, the study design had some shortcomings. Half of the AD patients were not blinded and knew which treatment they were receiving, and the significance reported for these measures reflected a within-group comparison (before vs. after treatment), as opposed to plasma vs. placebo. A handful of additional clinical trials, such as trials on the potential benefits of young plasma for AD and Parkinson’s disease (NCT03765762 and NCT03713957) and a study on the use of umbilical cord blood for treating age-related frailty (NCT02418013), are currently being performed or were recently completed. However, these studies are small and of limited duration. Often, they lack placebo controls, underlining their preliminary nature. Overall, to date, there is no real convincing evidence to support the effectiveness of young plasma as an anti-aging treatment in humans.

Unfortunately, the possibility of tissue rejuvenation by young plasma injection also raised the interest of some for-profit stem cell clinics and biotech companies, which started to transfuse plasma to humans according to scientifically dubious protocols regarding the amount, number, and timing of infusions. In February 2019, the FDA issued a general warning to consumers: plasma infusion from young people has not yet provided enough proven clinical benefit against aging and/or its diseases to justify its adoption on many individuals. One of these companies claimed that they did not need approval by the FDA because plasma transfusion is a well-established clinical practice and continued the treatments.

### Need for a suitable assay to identify the more efficacious plasmas

The results obtained to date by the injection of young plasma in aged rodents and humans, while confirming the great interest in the potential rejuvenation capability of this plasma, on the other hand underline the need for additional investigations before one can seriously think of a large-scale treatment of human subjects. Required investigations include the development of assays to measure the biological activity of the different human plasmas and to select the more efficacious plasmas.

We reported that in primary cultures of cells obtained from tissue biopsies, the platelet lysate activated signal transduction pathways distinctive of proliferation and was required for the re-entry of cells in the cell cycle, while the non-cellular component of the blood supported the cell division. On the contrary, in constitutively activated cell lines, plasma or plasma-derived serum sustained cell proliferation, regardless of the co-presence of the platelet lysate (Muraglia et al., 2017). Therefore, to be able to measure the capacity of plasma to induce cell proliferation appears highly relevant to assess the clinical potential of individual plasmas. We started from these observations to develop and propose a simple method to measure and compare the capability of different plasmas in supporting cell proliferation, thus determining their potential use or nonuse for clinical applications. The adoption of standardized cell lines and culture conditions and the identification of absolute indicator values should allow the direct comparison of unlimited plasmas also when tested at different times. The article is focused on the potential use of young plasma as a “rejuvenating” agent. However, the selection of more efficacious plasmas could also be important for the preparation of allogenic Platelet-Rich Plasma (PRP) to be used for the healing of different tissue damages, a therapeutic approach which has proven to be beneficial in several clinical trials.

## Methods

### Blood collection and plasma preparation

Plasma was derived from blood samples of young (age: 18–25 years; average age: 21.6 ± 1.8 years; 8 males and 12 females) and old subjects (age: 75–99 years; average age: 85.6 ± 6.1 years; 10 males and 9 females) obtained from the Transfusion Center and the Emergency and Geriatric Clinic of “Ospedale Policlinico” San Martino, Genova, Italy. A total of 39 plasmas were tested since one plasma was tested in both types of cell cultures. More specifically, plasmas from young subjects were collected from regular blood donors. All young blood donors were in good health, according to the criteria followed by the Transfusion Center, to allow blood donation. Blood donation is not allowed after 60 years of age. Therefore, the plasmas of the old subjects utilized were collected from voluntary donors and hospitalized patients. Plasmas to support the proliferation of MDA cells were obtained from the following subjects: three subjects from the emergency department because of a fall associated in one case to head trauma/presence of deep thrombosis/hypertension and subarachnoid hemorrhage; three subjects temporarily hospitalized in the geriatric clinic because of a mental illness associated with psoriasis with allergic asthma, diabetes, and acute heart failure, respectively; and four subjects hospitalized for a femur fracture. No significant differences were observed between plasmas from fallen subjects and the patients with mental illness. The old plasmas utilized for the HFFF2 cultures were from healthy voluntary donors (n = 2), fallen/fractured individuals (n = 6), and a patient with osteoporosis associated with intestinal perforation (n = 1). All patients were of Caucasian origin.

No specific clustering was observed in cell duplication values obtained from the patients of the different subgroups. All participants provided written informed consent for their participation in the study. Whole blood was harvested in K_3_-EDTA-containing sampling tubes and was centrifuged at 1,600 g for 15 min at 4°C. The supernatant, corresponding to the plasma fraction, was recovered, divided into aliquots and stored at −80°C until further use.

### Cell culture

Human serum from the whole blood, i.e., a combination of the platelet content and the non-cellular component of blood, was adopted as the cell culture medium supplement to replace fetal calf serum (FCS), with even higher efficiency. In constitutively activated cell lines, plasma or plasma-derived serum sustained cell proliferation, regardless of the co-presence of the platelet lysate (Muraglia et al., 2017). The human breast cancer-derived MDA cells maintained in culture in Dulbecco’s modified Eagle’s medium (DMEM) supplemented with 10% FCS were plated at a concentration of 1 × 10^3^ cells per well in 24-well plates. After three days of culture, cells were gently rinsed with phosphate-buffered saline (PBS), starved for 2 h in serum-free medium and then treated with fresh medium containing the tested plasma at the concentration of 0.1, 0.5, 1 and 2.5%. To avoid the activation of plasma clotting by cell-released proteases within the Petri dishes, 5 UI/mL sodium heparin was added to the medium. From the day of the medium change up to day 5 of the culture, the cells were detached daily by trypsin–EDTA digestion from triplicate wells of each plasma concentration series and counted.

Human Foetal Foreskin Fibroblast (HFFF2) cells suspended in DMEM containing 10% FCS were plated at a concentration of 2 × 10^3^ in wells of 24-well plates. The cells were plated in triplicate wells for each of the two chosen plasma concentrations (2.5% and 0.1%) and the two times to be tested (days 0 and 6). After 48 h from plating, the medium was removed, the cells were rinsed with PBS, then transferred to a serum-free medium for 1 h, and finally cultured with a fresh medium containing the plasma. Given the lower proliferation rate of the HFFF2 cell line than that of MDA cells, we stopped the cultures, and we determined the number of cell doublings 6 days from the change of medium instead of 5 days. MDA and HFFF2 cultures were performed at 37°C and 5% CO_2_.

### Protein analysis

To detect possible cytokine and inflammatory protein concentration differences between young and old plasmas, we used the Human XL Cytokine Array Kit (Catalog Number: ARY022B; R&D Systems, Inc., Minneapolis, United States), a tool used for the parallel determination of the relative levels of 105 selected human proteins. On each array, we tested plasma from both a young and an old individual. Loaded volumes of old and young plasmas contained 200 μg of proteins. The protein concentration was assessed by the Pierce™ BCA Protein Assay (Thermo Scientific). The protein signal was obtained by a UVITEC chemiluminescence imaging system and counted by ImageJ using the appropriate macro (Klemm, 2020).

### Statistical analysis

Differences between cell culture conditions were assessed by the non-parametric Mann–Whitney test. The protein signal differences were calculated by Student’s *t*-test. Statistical significance was accepted for any *p*-value ≤ 0.05. Analyses were performed using GraphPad Prism 9.4.0 software. Cytokine array data were analyzed using a custom R script including Bioconductor packages. In particular, log-transformed data were normalized by quantile normalization using the preprocessCore package. Then, differential enrichment analysis was performed using limma package (Ritchie et al., 2015). *p*-values derived from Bayesian statistics were adjusted by the Benjamini and Hochberg method.

## Results

In preliminary experiments, we observed that human plasma promoted the proliferation of several human cell lines, including HFFF2, HeLa, MDA, and U-937.

**FIGURE 1:**
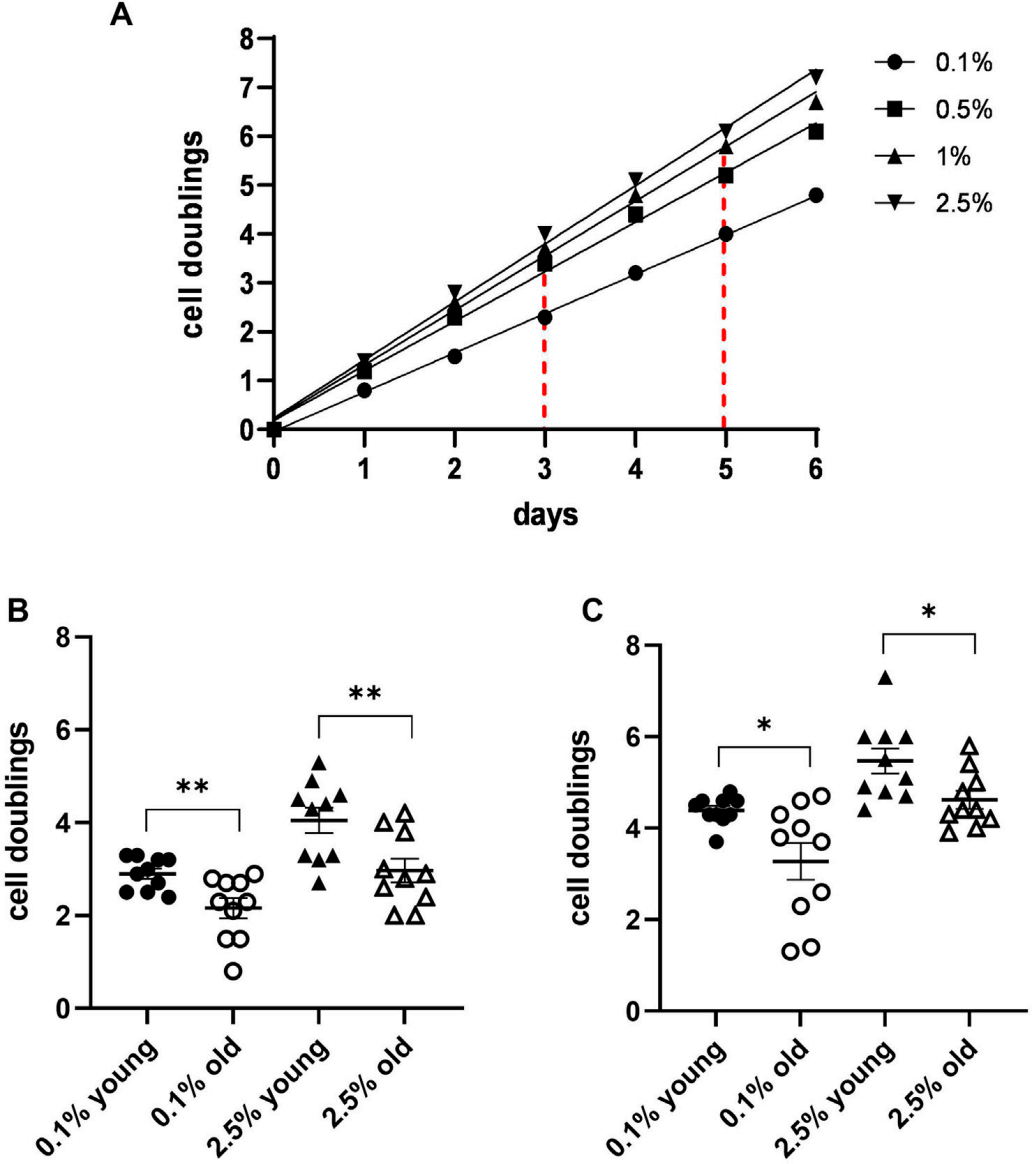
MDA cell line growth curves in the presence of different concentrations of plasma from a single blood donor. **(A)** Growth curves were obtained by cell doublings at different times from the culture start. The graph also shows how to determine values of two different indicators: number of duplications performed by the cells in 3 and 5 days in the presence of 0.1% and 2.5% plasma, respectively. **(B)** A scatter dot plot comparison of the indicators in a 3-day culture for 10 plasmas from young donors and 10 plasmas from subjects older than 75 years revealed significant differences between the two groups. **(C)** Scatter dot plot comparison as in **B** in a 5-day culture (*p ≤ 0.05 and **p ≤ 0.01).

An example of the obtained MDA cell proliferation at the different plasma concentrations is shown in Figure 1A. Based on the obtained cell doublings, we found indicators specific for each tested plasma concentration, i.e., number of cell duplications performed on days 3 and 5 in the presence of 0.1% and 2.5% plasma. A comparison of these indicators for 10 plasma concentrations from young subjects and 10 plasma concentrations from individuals older than 75 years revealed significant differences at 3 and 5 days (Figures 1B–C).

**FIGURE 2:**
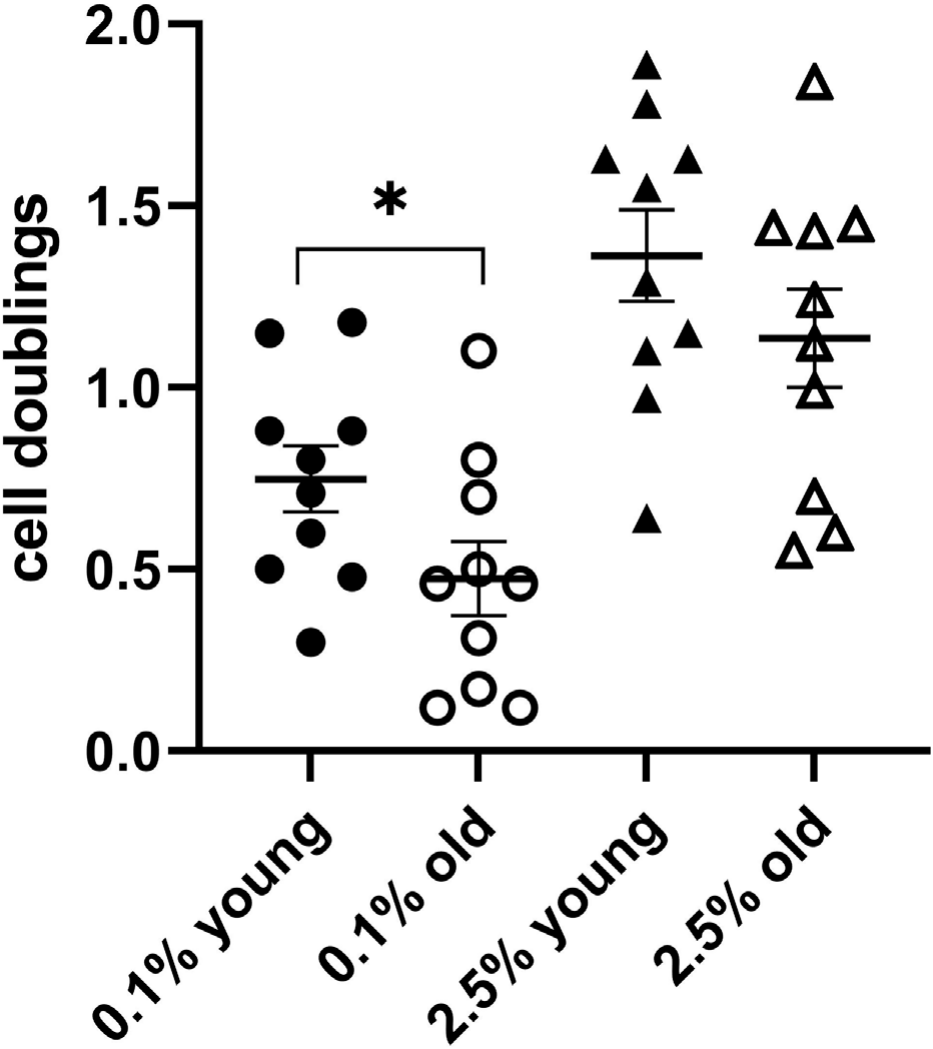
Test assay validation with HFFF2 cell line cultures. A scatter dot plot comparison of the indicators in a 6-day culture for 10 plasmas from young donors and 10 plasmas from subjects older than 75 years confirmed the differences between the two groups (*p ≤ 0.05).

Cell lines with a high proliferation rate tend to round up and lose cell adherence to the culture dishes at the time of their division. This could raise some difficulties when the cell counting is performed by scientists not particularly proficient in cell culture technologies. Therefore, we performed some assays also with cultures of the HFFF2 cell line, derived from a 14–18-week-old human fetus, with a longer duplication time. This cell line maintains a good cell adherence to the culture dish during proliferation and is easily available from cell bank repositories. At the time of medium change (day 0) and after 6-day culture, the cells in the corresponding wells were counted, and the number of doublings performed in 6 days at the two plasma concentrations (cell division indicators) was determined. We tested plasmas from 20 individuals, 10 young (18–25 years) and 10 old individuals (over 75 years). The scatter dot plots of the two indicator values in the two groups are shown in Figure 2. The average of duplications at 6 days in the presence of 2.5% plasma in the two age groups was 1.4 and 1.1, respectively, the slower proliferating group being the old plasma. For the second indicator, the average duplications at 6 days in the presence of 0.1% plasma were 0.8 and 0.5, respectively, with a significant difference (*p* ≤ 0.05), again, the slower proliferating being the old plasma group.

### Protein analysis

**FIGURE 3:**
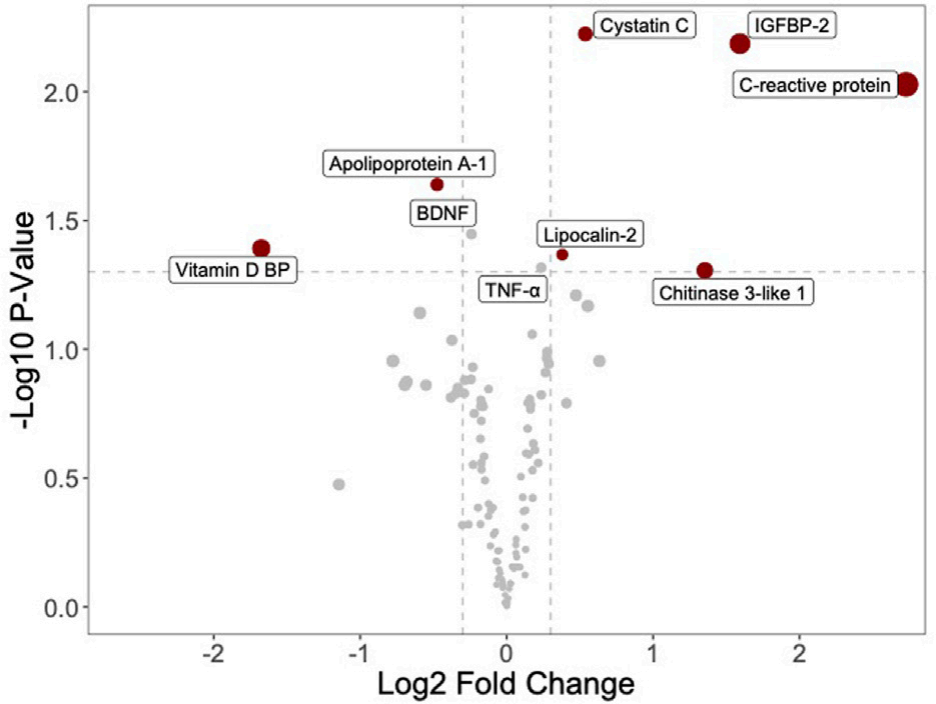
Volcano plot shows differential protein enrichment between young and old plasma samples. Proteins enriched in young plasma are displayed on the left, while those enriched in old plasma appear on the right. The analysis is confined to a selection of 105 proteins detectable by a commercially available protein array (Human XL Cytokine Array). Highlighted proteins display significant modulation (adjusted *p*-value <0.05).

On the assumption that the young blood effect may be attributed to specific proteins and growth factors more abundant in plasmas of younger individuals or the presence of inhibitors in the old plasmas, to detect possible cytokine and inflammatory protein concentration differences between young and old plasmas, we utilized four arrays from two different batches to analyze eight plasmas (four young and four old). Data were first analyzed by a volcano plot, a type of scatter plot that shows statistical significance (*p*-value) versus folds of change. All the data had a quite noisy distribution, so we used various pre-treatment approaches. We ended up by separately considering data from the two array batches, removing batch effects and then performing quantile normalization. The obtained volcano plot is shown in Figure 3. On the left side of the graph, one can see young plasma-enriched proteins, while on the right side are those enriched in the old plasmas. Statistically significantly expressed proteins are shown. Five proteins emerged as increased in the old plasmas: the C-reactive protein (CRP); an acute-phase protein produced by the liver; the circulating concentration of which increases in response to inflammation; the IGFBP2, a factor binding to insulin-like growth factors, thus allowing their free movement to target tissues; chitinase-3-like 1, a non-enzymatic protein playing a major role after a tissue injury; and two proteins synthesized by the cells in response to inflammation, cystatin C and lipocalin 2. Proteins present at a higher concentration in the young plasmas and decreased in the old were the vitamin D-binding protein, a multifunctional protein, that also binds to fatty acids and actin monomers, preventing their polymerization that could be detrimental to the circulatory system; and apolipoprotein A-1, an anti-inflammatory component of HDL, involved in cholesterol efflux.

**FIGURE 4:**
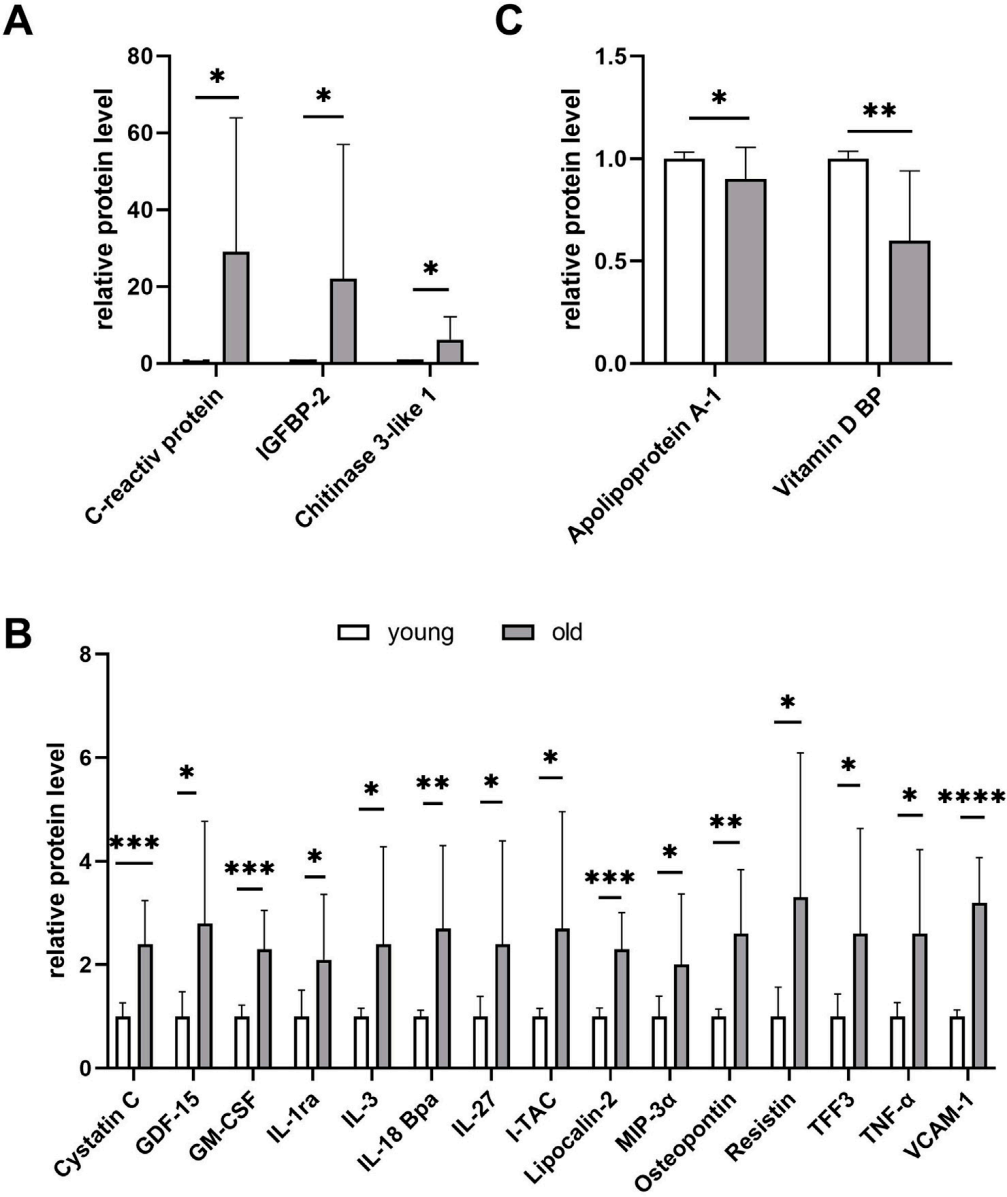
Proteome profile analysis of young (n = 4) and old plasma (n = 4) using the Human XL Cytokine Array Kit. The array considers 105 selected proteins in most cases correlated with inflammation. The graph summarizes the relative signal intensity of the significantly changed molecules. Shown are only proteins with a significant variation at the *t*-test (Student’s *t*-test, *p ≤ 0.05, **p ≤ 0.01, ***p ≤ 0.001, and ****p ≤ 0.0001). **(A)** Proteins overexpressed by more than 6-fold in old plasma compared to young plasma. **(B)** Proteins overexpressed by more than 2-fold in old compared to young plasma. **(C)** No protein showed a significant concentration decrease of at least 2-fold in the old plasmas compared to young plasmas. Shown are the only proteins with a significant decrease in the *t*-test.

As an alternative method of analysis, for each couple of plasmas (young and old) tested on the same array, we determined the fold increase or decrease in the protein concentration in the old plasma compared to the young plasma. Then, the results from the four different arrays were combined and the statistical significance evaluated. A summary is shown in Figure 4. The proteins with the highest concentration increases observed in the old plasmas were again CRP (approximately 30-fold increase, *p* = 0.03), IGFBP2 (approximately 20-fold increase, *p* = 0.01), and chitinase-3-like 1 (more than 6-fold increase, *p* = 0.02) (Figure 4A). More proteins significantly increased (between 2 and 4-fold, *p* = less than 0.04) in old plasmas, in addition to the already identified cystatin-C and lipocalin 2, including several cytokines involved in inflammation such as tumor necrosis factor alpha (TNF-α), with pleiotropic effects on various cell types, GDF-15, first identified as macrophage inhibitory cytokine-1, granulocyte macrophage colony-stimulating factor (GM-CSF), IL-1ra, IL-3, IL-18Bpa, also known as interferon-inducing factor, IL-27, interferon-inducible T-cell alpha chemoattractant (I-TAC), and macrophage inflammatory protein-1 alpha (MIP-1alpha/CCL3). Other proteins that increased in old plasmas and were synthesized in response to inflammation were osteopontin, resistin, and vascular cell adhesion molecule 1 (VCAM-1) (Figure 4B). Considering the increase in the old plasmas of these proteins related to inflammation, one could hypothesize a role for them as inhibitors of the plasma cell proliferation stimulatory activity. However, the coincidence of the increase in the inflammatory cytokines and proteins and the decrease in the cell proliferation induction does not necessarily imply a cause–effect relationship. Note the extreme variability in the concentration of these proteins in the plasmas of old subjects compared to young subjects, suggesting major concentration variations in the plasmas from different old individuals. Possibly, because of a bias in their selection, among the 105 proteins present on the array, none showed a significant decrease of at least 2-fold in the old plasmas. The only proteins with a significant decrease revealed in the *t*-test were the vitamin D-binding protein and the apolipoprotein A-1 (Figure 4C). However, the observed decreases were limited to 42% and 13%, respectively.

## Discussion and conclusions

The advantage of a replacement in human cell culture media of the bovine serum with human serum or plasma is well known. However, even though a selective beneficial effect of plasmas from young animals was observed *in vivo* in animal models and *in vitro* in cultures of animal cells (see Introduction), information about the efficacy of young human plasma compared to old plasma is scarce. Several papers showed that human serum from old and young subjects promotes cell proliferation at a similar extent in cells such as the human lung and skin fibroblasts (Kondo et al., 1988), tendon cells (Bayer et al., 2012), and myoblasts (George et al., 2010). In a lower number of cases, the human supplement was plasma. Kalampouka I et al. showed that old and young human plasma from single individuals had a similar effect on the proliferation of the murine skeletal muscle cell line C2C12 (Kalampouka et al., 2018). The efficacy of pooled human plasma from children and old donors was compared in human young and middle-aged-derived hematopoietic progenitor cells, showing that old plasma was more effective in inducing cell proliferation in middle-aged-derived cells, while young plasma was more effective in inducing proliferation of young donor cells (Santos et al., 2008). Interestingly, investigations on cell migration showed that, compared to young plasma, human old plasma was inhibitory on C2C12 cells (Kalampouka et al., 2018) and human fetal lung fibroblasts (Kondo et al., 1989). It is worth mentioning that in PRP derived from whole blood of 6 young and 6 elderly volunteers, after the removal of 95% of high-abundance proteins (albumin, globulins and fibrinogen), Jiang et al. identified a total of 739 proteins by mass spectrometry analysis (Jiang et al., 2024). Of these proteins, 76 were upregulated proteins in the young group and 235 upregulated in the elderly group. Proteins upregulated in the elderly subjects were associated with cell apoptosis, angiogenesis, and complement and coagulation cascades, whereas IGF-1 was upregulated in the young group, suggesting a possible role in the enhancement of cell proliferation. However, the analyzed samples also included proteins derived from the lysis of platelets, and the observed differences cannot be directly attributed to differences in the plasma proteins.

With our work, we did not want to confirm the already published data. Our main goal was to adopt an assay that could be utilized to determine the cell proliferation support activity of single plasmas. In the Introduction, we already explained the difference between human sera or plasmas containing platelet lysate, as in most published articles, and the human plasmas depleted of platelet lysate we propose. In fact, the platelet lysate plays a major role in cell commitment and re-entry from the G0 to the G1 cell cycle phase but cannot support cell proliferation. On the contrary, the liquid plasma promotes and supports cell proliferation but is unable to induce the re-entry of quiescent cells in the cell cycle (Muraglia et al., 2017). No major differences exist in the activity of platelet lysates from different blood donors (our unpublished results), whereas noteworthy variabilities exist in cell proliferation support activity of liquid plasmas. The use of plasmas depleted of platelet lysate allows the implementation of standardized cell lines and should eliminate variabilities associated with primary cultures.

In preliminary experiments, we tested different cell lines, including HFFF2, HeLa, MDA, and U-937, and obtained better results with plasmas from young compared to elderly subjects. More extended experiments were performed with MDA and HFFF2 cell lines characterized by different duplication times. Based on the results presented in this paper, we propose to use the MDA line for the plasma activity testing. However, each laboratory could decide to use the cell line more suitable for them, provided that in the future, the comparison of plasmas will be made always adopting the same cell line.

To validate the assay, we compared plasmas from young and old subjects and observed the expected decreased activity in those treated by plasmas from old individuals. It remains to be investigated whether this is the result of a higher concentration of specific growth factors and activators in the young plasma, or of the accumulation of inflammatory inhibitor molecules in the old plasma, or both. Some scientists attempted to identify and isolate specific factors responsible for the rejuvenating effects. These factors could decrease with age and contribute to the age-related decline in the body repair and regeneration ability. A protein called Growth Differentiation Factor 11 (GDF11) that is more abundant in younger than in older plasma could be an example of these proteins and factors (Añón-Hidalgo et al., 2019). Alternatively, or at the same time, the reduced promotion of the cell proliferation could be the result of the presence of inhibitors in the old plasma. An important feature of many senescent cells is the senescence-associated secretory phenotype (SASP). The SASP includes several inflammatory cytokines, chemokines, growth factors, and proteases, and it has been proposed to play a key role in inducing the deleterious effects of senescent cells by promoting aging. However, depending on the timing and the existing microenvironment, SASP could also have some beneficial effects, such as a stimulation of the immune clearance of senescent cells and the optimization of the repair of damaged tissues (Tchkonia et al., 2013). Circulating SASP factors are associated with advanced age. A panel of 24 SASP proteins identified *in vitro* was measured in the plasma of a random sample of 267 Mayo Clinic Biobank donors (Olson et al., 2013). The sample was equivalently distributed by sex and age from 20 to 90 years. Circulating concentrations of 19 SASP proteins were enriched in the plasma of old subjects, and the associations between 17 SASP factors and chronological age remained significant after adjusting for sex and body mass.

Although studying the different protein contents of plasmas from young and old individuals was out of the scope of this article, and such differences could be satisfactory investigated only by proteomics on a larger number of samples, we decided to verify whether there was an enrichment of inflammatory proteins in the old plasmas. We could show that significant protein concentration differences between young and old plasmas existed and that plasma from old individuals presented a higher concentration of “inflammation-related” proteins. An increase in the serum level of the five proteins whose concentrations in the volcano plot analysis were more abundant in old plasmas was also recently reported by other authors. In particular, i) studies investigating the association between aging and a state of chronic low-grade inflammation showed higher levels of inflammatory markers in serum, including CRP and TNF (Michaud et al., 2013). It was proposed that the increase in the inflammatory markers could be the result of a higher amount of adipose tissue, decrease in sex hormones, increased oxidative damage, and age-related diseases (Singh and Newman, 2011); ii) CHI3L1, encoding chitinase 3-like 1, was identified as the gene with the greatest age-dependent expression increase in liver tissue (Nishimura et al., 2021); iii) in what is probably the first study investigating the relationship between IGFBP-2 levels and age in a longitudinal setting, it was found that the serum IGFBP-2 level increases with age after the age of 50 years, evolves in parallel with insulin sensitivity, and may be a potential marker to predict mortality in the aging population (van den Beld et al., 2019); iv) the recent availability of cystatin C measurements as an alternative measure of kidney function allowed us to characterize cystatin C concentrations in more than 18,000 individuals aged 28–100 years. Across the age range, there was a strong, non-linear association of age with cystatin C concentration (Pottel et al., 2023); and v) serum LCN2 levels were correlated with age and bone turnover biomarkers in both healthy women and men (Maurizi et al., 2021). Although an inflammatory microenvironment is often associated with an increased cell proliferation, some of the circulating SASP factors we reported to be more abundant in the old plasmas are considered to be involved in the control of replicative senescence, as reported by Kumari R., underlining the link between a pro-inflammatory milieu and the age-dependent effects at the cellular level (Kumari and Jat, 2021).

In conclusion, to determine the potential use or nonuse of different plasmas for clinical applications, we proposed a simple assay to measure the potential of plasmas in supporting the proliferation of cell lines, regardless of the co-presence of a platelet lysate. Although the relatively small number of tested plasmas could be a limitation to the study, by comparing plasmas from young and old subjects, we observed a decreased activity in plasmas from old individuals, possibly due to the enriched presence of “inflammatory” molecules.

## Data Availability

The raw data supporting the conclusion of this article will be made available by the authors, without undue reservation.
